# Overview of Electricity Transmission Conductors: Challenges and Remedies

**DOI:** 10.3390/ma15228094

**Published:** 2022-11-15

**Authors:** Chika Oliver Ujah, Daramy Vandi Von Kallon, Victor Sunday Aigbodion

**Affiliations:** 1 Department of Mechanical and Industrial Engineering Technology, University of Johannesburg, Johannesburg 2006, South Africa; 2 Africa Centre of Excellence for Sustainable Power and Energy Development (ACE-SPED), University of Nigeria, Nsukka 410001, Nigeria; 3 Faculty of Engineering and Built Environment, University of Johannesburg, Johannesburg 2006, South Africa; 4 Department of Metallurgical and Materials Engineering, University of Nigeria, Nsukka 410001, Nigeria

**Keywords:** transmission conductor, extrusion, pultrusion, spark plasma sintering, aluminium conductor, high-temperature low-sag conductors

## Abstract

Electricity transmission is an essential intermediary linking power generation and distribution. Voltage drops or total blackouts have always characterized the transmission and distribution of electricity in the sub-Saharan Africa and some Asian dwellers. This has been attributed partly to faulty, defective or dilapidated transmission conductors/networks. The aim of this study is to identify the causes of those defects in the transmission conductors and proffer possible remedies to them. Studies have shown that the current production techniques of transmission conductors (TCs) generate defective products, and that the materials used have their own challenges too. This work, therefore, reviewed all the production techniques and materials used in the development of TCs. It was observed that pultrusion, extrusion, hot-rolling, and stir-casting were the techniques used in the production of transmission conductors. Defects such as shrinkage, pores, impurities, and warps were identified in those techniques and some recommendations to ameliorate the defects of those techniques were presented. Spark plasma sintering is recommended as the most promising solid- state production techniques that should be adopted in fabricating transmission conductors, though it is yet to be developed for producing long-span products. In addition, advanced TCs materials such as Al-CNTs, Al-Nb, Al-Ti, and Al-B2 were presented as better alternatives to the existing TCs materials. By producing TCs with the recommended techniques and materials, the electricity availability will be enhanced; and this will lead to sustainable industrial growth and economic stability in the third world countries and the entire world.

## 1. Introduction

Electricity transmission is the necessary intermediary linking electricity generation and its distribution to the consumers. It is only efficient and effective electricity transmission that ensures high power delivery to target areas. Poor transmission of electricity or drop in voltage across transmission lines has been attributed to a number of factors which comprise those contributed by the nature of the materials used in the development of the conductor and those contributed by the development technique. Electricity transmission loss results in an epileptic power supply or total black out. Research shows that one of the factors contributing to power transmission loss is electrical treeing. This is caused by impurities entrapped in the conductor material and/or mechanical defects imposed on the conductor during the installation, such as abrasion. Electrical treeing manifests as partial discharges or sparks on the conductor when current flows across a portion of the conductor harbouring the entrapment [1]. These sparks appear in a tree-like configuration and result in a voltage drop or severe damage to the transmission line. The extreme case of electrical treeing leads to total burning down of the transmission line. The panacea to this defect is the usage of an advanced production technique which produces impurity-free products and the application of advanced hybrid/nanomaterials with high resistance to abrasion and corrosion. Meanwhile, one of the most outstanding conventional transmission conductors is Aluminium Conductor Steel Reinforced (ACSR). Its efficiency in power transmission is affected by the high density of its steel core, high affinity to corrosion of the steel core, and high coefficient of thermal expansion (CTE) of steel materials. These factors limit its current-carrying capacity (ampacity) as well as its cost effectiveness [2,3,4]. So, the steel core needs to be replaced with advanced light materials with higher corrosion resistance, higher wear resistance, and lower CTE. It will be recalled that the coefficient of thermal expansion of composite materials is dependent on the thermal conductivity of the constituent elements [5] which then determines its sag level when current traverses the transmission line as well as its ampacity. More so, the aluminium conductor composite reinforced (ACCR) is another high-performing transmission conductor in the market. However, Banerjee [6] disclosed that the CTE of its metal matrix core (MMC) is relatively high, measuring about 6 × 10^−6^ K^−1^, which makes it susceptible to high sag. This author also revealed that the polymer matrix composite (PMC) used in the production of the aluminium conductor composite core (ACCC), which is another high-performing transmission conductor, can only perform optimally at temperatures below 125 °C, after which degradation ensues. The implication of this is that advanced composites, hybrid or refractory materials need to be used to replace the polymer core that is susceptible to thermal degradation at elevated temperatures. Some recent works on the development of high-temperature, high-strength, and corrosion-resistant materials were recently reported [7,8]. One of the developed materials consisted of an outer wire made of an Al-Nb alloy and the inner core made of an Al-CNTs-Nb composite. The electrical conductivity, thermal conductivity, tribological characteristics, corrosion resistance, and mechanical strength of the composite conductor material were superior to the available high-temperature low-sag (HTLS) conductors. However, it was observed that Nb is a high-density element, and the density would increase the density of the TC, which impacts negatively the conductor. Therefore, a lighter-weight material with more robust characteristics was recommended by the authors. It will be noted that density plays a vital role in transmission conductors because it determines the number of pole supports needed to carry the TC in the grid. Denser conductors require more pole supports which translates to higher cost. There are many works on transmission conductors in the literature. However, no attention has been given to the production technique as one of the causes of the grid crisis. More so, little attention has equally been given to the need to replace the existing transmission conductor materials with the emerging nanomaterials. In this review, effort was made to review the existing production techniques of transmission conductors, the materials used in the development of existing transmission conductors, and proffer better production methods and materials that would enhance transmission of electricity more efficiently, more effectively, and optimally at cheaper rate.

## 2. Strengths and Weaknesses of Transmission Conductors 

Transmission conductors come in various forms and shapes. Materials used in the production of transmission conductors are numerous. In this section, those materials are discussed, showcasing their strengths and weaknesses. Magnetic properties of materials have negative effect on the electrical conductivity of materials because electrons are repelled from each other for onward transmission/conduction of current. Hence, materials that possess low magnetic properties are required for the development of electrical conductors so that a repulsive force will be of high value. Among all metals, silver has the highest electrical conductivity of 100 on a 0 to 100 scale ranking while copper and gold are ranked 97 and 76, respectively. However, Cu is less expensive and Au is more corrosion-resistant and that is why they are more often applied in electrical conductors than Ag. It is because of the cost effectiveness that Cu became more popular than the other two highly conductive metals. For this reason, Cu also became the international standard conductor to which other conductors were measured. That is why it is called the International Annealed Copper Standard (IACS) from which other electrical conductors are referred. The reference was adopted in 1913 where annealed copper (Cu) was assigned the electrical conductivity of 100% IACS. Till date, Cu cables have been applied in electrical installations of buildings, electrical and electronic gadgets; while Cu-Cu windings are used in power transformers [9,10]. Lloyd and Clement [11] opined that high density, vulnerability to corrosion, lack of passivation oxides, and its inclination to attacking silicon junctions in electronics have undermined the usage of copper (Cu) cables in electrical applications. Then, the interest shifted to Al conductors as a better replacement for Cu in electrical conductors. Aluminium (Al) has an electrical conductivity range of 21–63% IACS where the conductivity depends on the type of Al and heat treatment it is subjected into. 

### 2.1. All Aluminium Transmission Conductors

**Figure 1:**
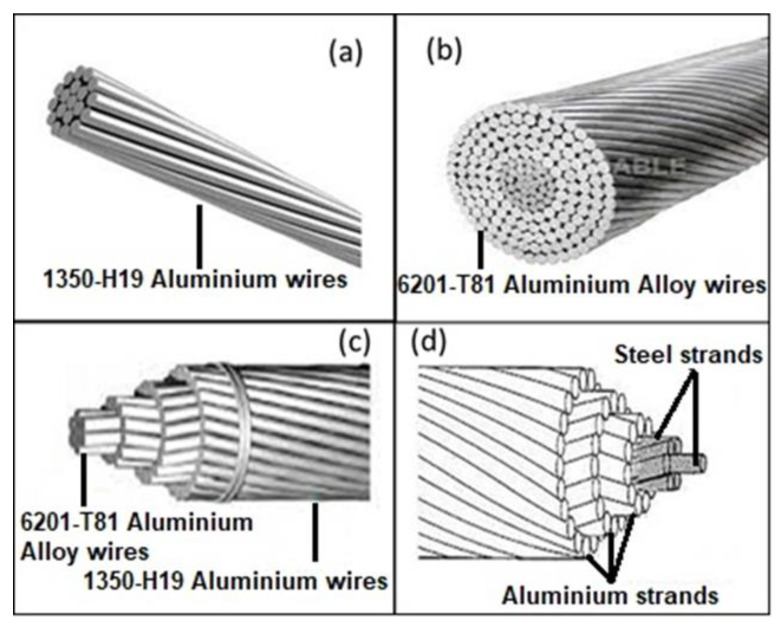
Al Transmission Conductors (**a**) AAC, (**b**) AAAC, (**c**) ACAR, (**d**) ACSR [12].

Presently, there are about four major types of Al conductors, namely: aluminium alloy conductor (AAC), all-aluminium alloy conductor (AAAC), aluminium conductor alloy reinforced (ACAR), aluminium conductor steel reinforced (ACSR) as shown in Figure 1. 

AAC consists of many strands of hard-drawn 1350-H19 tempered Al alloy with minimum electrical conductivity of 61.2% IACS. This conductor is utilized in municipal electricity distribution that possess limited spacing and closely positioned supports [4]. Its corrosion resistance is appreciable and so used in coastal regions where ice and dews are salty. Meanwhile, it has low strength, and because it uses closely positioned supports, it is not cost effective. These challenges have undermined its use as a transmission conductor. Moreover, AAAC, according to Hesterlee, Sanders [13] consists of 6201-T81 aluminium alloy which enjoys high corrosion resistance of AAC together with high strength of heat-treated Al. It is applied in power transmission/distribution that requires sparsely located supports such as in valleys and rivers crossing. In addition, it is utilized where there is a corrosion challenge. Its conductivity is about 52.5% IACS which is below that of AAC [4]. The third type of Al conductor is the aluminium conductor aluminium reinforced (ACAR) which enjoys the high electrical conductivity of 1350 Al alloy and high strength of 6201 Al alloy to produce a stable conductor of high strength and excellent electrical conductivity [4]. It is made up of several layers of 1350-H19 aluminium strands wrapped on 6201-T81 aluminium wires, with a central core made of 6201 Al strands. The flexibility of the design is in such a way that both the outer strands and the core can be interswitched so as to satisfy the demand of the area of application. As 1350-H19 serves as the core and 6201-T18 the outer surface in one region, they can be interchanged in another region based on the demand of the area [13]. The most versatile Al conductor is the aluminium conductor steel reinforced (ACSR). Hesterlee, Sanders [13] described ACSR as the traditional transmission conductor which came into use since 1900, and consists of stranded galvanised steel central core enclosed by layers of 1350-H19 Al wire. The quantity of steel in the core determines strength of the conductor and the quantity of steel incorporated in a typical ACSR is in the range of 7–40%. Its application is in very long span crossing such as long rivers and hills. The strength of this conductor is the steel core which equips it to withstand more ice and wind loads. Its ampacity is higher than other all-Al conductors but the sag level is greater. Unfortunately too, the steel core has high density which impacts negatively to the cost of using this TC. In addition, the steel core is highly susceptible to corrosion [2]; while its maximum operating temperature is between 95–100 °C. It is due to these challenges identified in the all-Al conventional conductors that high-temperature low-sag (HTLS) conductors were developed. Alawar et al. [14] opined that as the demand for electricity kept surging at a rate of 25% per decade, while electric transmission facilities are upgraded or maintained at a rate of 4% per decade, that it will be imperative to invent more robust transmission conductors with more durability, and higher ampacity. 

### 2.2. HTLS Transmission Conductors

One of the interesting characteristics of HTLS conductors is that it can be mounted in an existing Al or Cu transmission line and give double of its ampacity; thereby reducing cost, time, and power outage which would have occurred during conventional upgrading of transmission lines. So, HTLS conductors were invented to be used in replacing traditional aluminium or copper conductors in the grid without elaborate modifications. They can operate between 100 °C and 250 °C with very minimal sag and low loss of strength. Their benefits include saving time, minimal labour and low cost [15,16,17]. Types, material properties and deficiencies of HTLS conductors are shown in Table 1. The essential properties of HTLS conductors cannot be overemphasized. However, there are still some challenges ravaging them which are itemized in Table 1. For example, ACCR is a high-performing transmission conductor but has relatively high CTE which affects its sag level; and its core which is alumina fibre is consolidated with extrusion. This extrusion technique is liable to contaminate the conductor with cracks, piping and impurities [18] which can result in the treeing defect witnessed in transmission lines. Thus, the functionality of the TC is usually impaired. Moreover, ACCC is another highly rated transmission conductor but is ravaged with low strength; and its operating temperature is relatively low (130 °C) [6]. The production technique is pultrusion which is susceptible to contamination, warped shape, and irregular cross-section [19]. Studies show that all other steel-based HTLS conductors have high density and susceptible to corrosion. Therefore, it has been seen that the materials applied in the existing transmission conductors, both all-Al conventional conductors and HTLS TCs have deficiency in one way or the other. These deficiencies affect their efficiency, ampacity, durability, and cost effectiveness; and so, constitute the bane of the power grid. Hence, there is the need to research on more robust techniques and materials with more robust characteristics. 

## 3. Strengths and Defects of Production Techniques Used in Transmission Conductors

Production techniques employed in the development of transmission conductors include hot-rolling, extrusion, pultrusion, and stir-casting. All these techniques have their inherent challenges which are discussed in this section. 

### 3.1. Hot-Rolling

**Figure 2:**
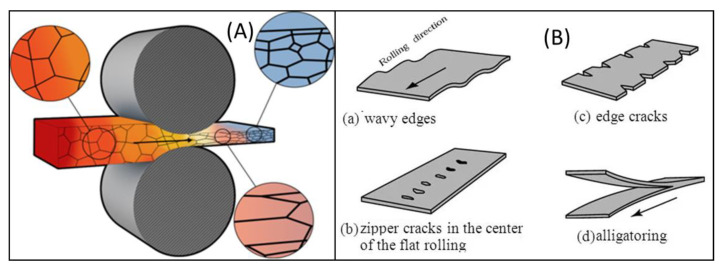
Image of Hot-Rolled Composite Sample Showing Alteration in Microstructure and the Physical Geometry [29]. (**A**) Alteration in Microstructure and Physical Structure, (**B**) Defects Associated with Hot Rolling Technique.

Hot-rolling is a metal fabrication process whereby metal is heated above the recrystallization temperature so as to plastically deform it by the replacement of defect grains with defect-free grains. This technique is used to produce shapes with preferred geometrical sizes and material characteristics without alteration of its volume. Here, the process affects the internal microstructure, the physical configuration, and the shape (Figure 2). It is employed in the production of most of monolithic transmission conductors such as Al and Cu conductors. Hot-rolled conductors are challenged by both surface-rolling defects and internal structural rolling defects such as wavy edge crack, zipper crack, alligator crack, and edge cracks (Figure 2B) [27,28]. 

Despite the challenges associated with hot-rolling, it enjoys a plethora of advantages. In a work to determine the effect of hot-rolling on a fabricated Ti-15Mo/TiB metal matrix composite, Zherebtsov et al. [30] discovered that metastable α″ and isothermal ω phases in the fabricated composite changed to more stable α phase after hot-rolling. There was a 12% improvement in ductility in comparison with a non-hot-rolled composite. The average grain size of TiB decreased from 400 ± 200 nm to 12 ± 6 nm, while the whisker decreased from 5 ± 2 μm to 3 ± 1.2 μm. The only negative effect was that the yield strength decreased from 1360 MPa to 1330 MPa after hot-rolling. The result indicates that hot-rolling alters the microstructure, decreases strength and hardness but increases ductility. Liu et al. [31] developed CNTs/Al via friction stir processing and hot-rolling and discovered that the ultimate tensile strength, yield strength, ductility and microstructure of the friction stir processed and hot-rolled composite were better than that produced without hot-rolling. Yin et al. [32] stressed that even though hot-rolling improved mechanical strength and elongation, it provokes enormous deformation at a higher temperature which affects the homogeneity of the microstructure. Lartigue-Korinek et al. [33] developed Fe-TiB2 using hot-rolling and observed that plasticity occurred in the TiB_2_ phase which contributed to a low deformation damage of the composite. In comparison of stir-casting and hot-rolling of Al-ZrB_2_ in situ composite, Kumar et al. [34] discovered that the hardness of as-cast and as-hot-rolled composite became improved by 18% and 29%, respectively; while their ultimate tensile strengths were almost the same with identical grain refinement. However, during hot-rolling, surface defects upsurged as the friction acting in between the roll and strip increased [35]. The red-scale defect observed in hot-rolled Si steel can be descaled through hydraulic descaling if and only if the Si content is low but when the Si content is high, it becomes a permanent defect in steel-rolling [36]. Wang et al. [37] identified five types of edge defects obtainable in hot-rolling to include upwarp, black line, crack, slag inclusion, and gas hole. Seam defect, blisters, and slivers were severe hot-rolling defects which affected the painted appearance, structural integrity, durability, and performance of hot-rolled products [38,39]. Various characteristic defects observed in hot-rolling according to Utsunomiya et al. [27] included uniform deformation with matrix material, cracking, fragmentation, and indentation to matrix material; but posited that the scale defect can be ameliorated by ensuring that the scale before rolling should be thinner than the critical thickness which is dependent on the rolling temperature. By and large, it has been confirmed that hot-rolling is a useful tool for the development of metal matrix composites because of the essential properties it bequeaths on its products but possesses numerous surface and internal defects which militate against the maximum functionality of its products. Therefore, more robust ways of preventing the defects of hot-rolling are recommended to be researched. 

### 3.2. Extrusion

**Figure 3:**
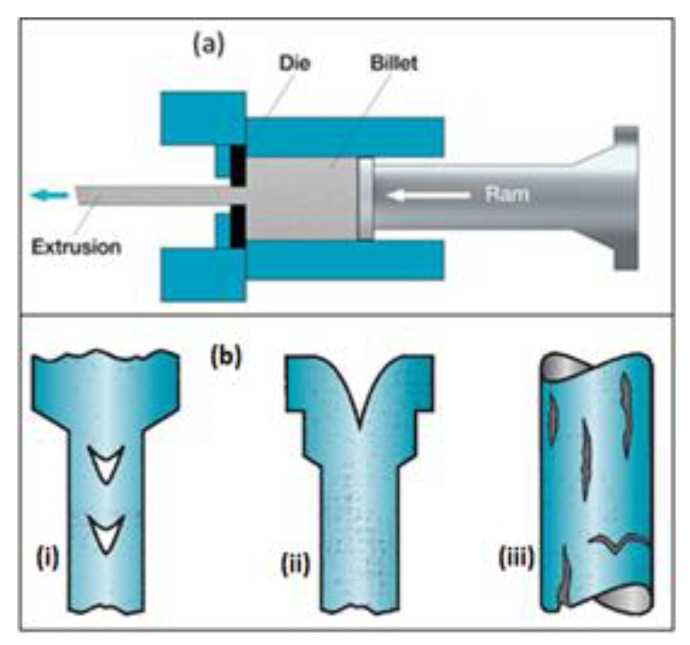
(**a**) Extrusion Process, (**b**) Common Defects in Extrusion, (**i**) Central Burst, (**ii**) Piping, (**iii**) Surface Cracking [52].

Extrusion is another conventional transmission conductor fabrication technique employed in the production of aluminium conductor composite reinforced (ACCR) [40]. It is a consolidation technique in which materials such as ceramics, composites, and plastics are converted from solid to liquid and vice versa without compromising their inherent characteristics. If the material is solid, it is melted and pushed through an orifice which shapes it into a desired cross-section and configuration. The factors considered during extrusion, according to Thomas [41], include screw geometry, screw rotation speed, and barrel heater. The benefits of extrusion include reduced cost, easy of handling, flexible to shape alteration; while the defects include shape and size increase, restrictions to products that can be extruded. Skorpen et al. [42] were able to achieve enhanced microstructure and mechanical properties with extrusion; while Kuzumaki et al. [43] produced an Al-CNTs composite devoid of detrimental Al_3_C_4_ intermetallic with extrusion. Kwon et al. [44] developed Al-1vol.% CNTs with ball-milling and hot extrusion and achieved an improved hardness and tensile strength of about three times in comparison to unalloyed Al even though there was presence of traces of Al_3_C_4_ intermetallic. Dvorský [45] observed an improved corrosion characteristics of magnesium fluoride (MgF) composite prepared via extrusion method. Extrusion-based additive manufacturing (AM) is an advanced extrusion technique whereby a computer-controlled layer-by-layer deposition of molten and semi-molten polymers, pastes, solutions, and dispersions via a mobile jet acting as the extrusion print head is achieved [46]. This technique is cost effective, has rapid manufacturing capacity and minimal waste, together with ability to printing of complex 3D structures [47]. One of the forms of this method is called fused filament fabrication (FFF) which is a slurry extrusion-dependent technique that melts solid filaments via a heated nozzle to produce layer-by-layer complex structures from Computer-Aided Design (CAD) [47]. FFF makes use of a thermoplastic matrix because it can be melt-extruded at or beyond the glass transition temperature via a heated extruder. Another extrusion-based AM is direct ink writing (DIW). It has a larger scope than FFF because it can work on thermosets, thermoplastics, metals, ceramics, and cements [48]. Sarvestani et al. [49] worked on the structural responses, failure kinetics, and energy absorption potentials of FFF structures with various core configurations. It was observed that the core topology and geometric constraints of the meta-sandwich structures were essential to their failure mechanism and energy absorption potentials. Structures such as isomax, octet, and cubic meta-sandwich possessed higher energy absorption than the auxetic core for low impact energy, whereas octet meta-sandwich structures had better performance at higher impact energy. Meanwhile, Arif et al. [50] identified a number of defects accruing from extrusion method as follow: faulty billets which includes slag/impurity addition, scales/flakes, internal fissures, oxide inclusions; inappropriate tooling which includes billet and die preheat furnaces, dies/mandrels and dimensional correction tool; defects from extrusion operation which includes unsuitable extrusion pressure leading to surface cracking (Figure 3b(iii)), inadequate chamber temperature, excess friction, high ram speed; faults from post-extrusion operations such as saw cutting, stretching/straightening, roll correction, age hardening, anodizing, and painting. Ko et al. [51] observed another defect called central burst defect (Figure 3b(i)) in extruded products which is very difficult to detect through ordinary inspection. 

It was also observed that a zigzag surface experienced in extruded products is a result of excessive shear stress at the nozzle wall or the interface while parabolic or finger-like structures are a result of flow disturbances [53]. Khan [18] identified more than ten defects experienced in extrusion as shown in Table 2. So, from the literature, it can be seen that extrusion has good number of benefits but is bevelled with plethora of defects. So, improved alternative techniques such as FFF and DIW need to be developed further into commercial standard for its application in the manufacture of TCs. However, if the conventional extrusion method must be used, appropriate precautionary measures as captured in Table 2 must be followed. 

### 3.3. Pultrusion

**Figure 4:**
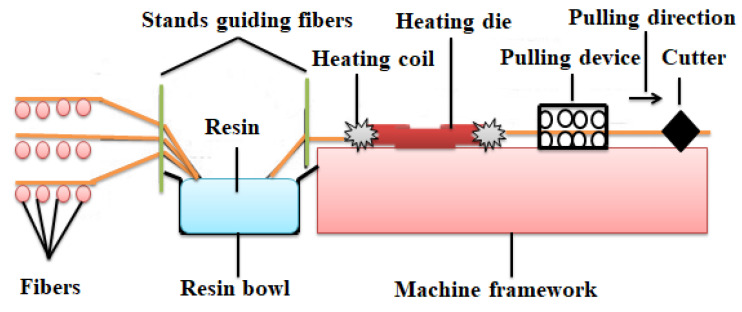
Image of Pultrusion Process Adapted and Redrawn from [61].

Pultrusion is another conventional production method whereby reinforcement is positioned longitudinally in a device and saturated with resin and pulled through a heated orifice and formed to a preferred configuration (see Figure 4). It is the technique adopted in the production of an aluminium conductor composite core (ACCC) conductor [14]. Pultrusion was developed in the 1950s by W. Brant Goldsworthy but was patented only to be used in producing fishing rods [54]. Its products have essential characteristics which include high strength, high endurance, high corrosion resistance, low density, high installation flexibility, with low maintenance requirement [41]. Chandrashekhara et al. [55] opined that pultrusion has a high level of superiority over other techniques because the reinforcements (mostly fibres) are drawn under tension in the nozzle which in essence promotes its strength. However, they added that pultrusion is ravaged by irregular cross-section of products, high energy requirement, and high cost. Nosbi et al. [56] pointed out that the major benefit of pultrusion is its high stiffness stimulated by high fibre absorption ability. It was observed that the major processing parameters in pultrusion included resin viscosity, fibre content, orifice temperature, resin polymerization, and pulling speed; but the orifice temperature was the most essential parameter which dictates the structure of the pultruded product since irregular heat dissemination breeds irregular curing which produces warped products [57]. Baran et al. [58] opined that the deformation of pultruded products is a result of volume contraction of the resin by the die and not by thermal expansion and contraction since the temperature gradients of the orifice and resin is very small. Krasnovskii and Kazakov [59] advised for a uniformity and slowness of the pulling speed of the pultrusion machine so as to hinder cracks promoted by swift and non-uniform pulling; which breed fibre breakages or warped products in extreme cases. Giordano and Nicolais [60] noted that the quality of pultruded products can be improved by guiding the polymerization and rheological kinetics of the resin. So, from the above discussion, it can be seen that pultrusion has a good number of challenges. The incessant heating of die is not cost-effective. Pultruded products are not regular in shape and size; they are usually warped. Non-uniformly cured resin generates cracked and deformed products. This technique has no impurity inspection device. However, these challenges can be ameliorated when adequate preventive measures itemized above are taken. 

## 4. Recent Advancements in Production of Composite Systems 

Studies show that composite materials are the most promising materials for transmission conductors. Therefore, when production routes of metal matrix composites (MMC) and polymer matrix composites (PMC) are being discussed, production routes of TCs materials are being discussed as well. So, in this section, the latest advances in production of MMC and PMC are discussed. It is imperative to know that different production routes can produce absolutely different composites despite that the same “as-received” matrix and reinforcements with similar composition are used for the production [62]. This implies that production routes can improve or degrade material properties. Production of MMC and PMC can be categorized into primary and secondary production processes. The primary process entails blending the constituent materials and consolidation, while the secondary process involves shaping or joining of materials [63]. Another categorization is based on how the dispersed phases are introduced into the matrix or base material. If the dispersed phase is created within the composite through chemical reaction which is usually exothermic, the production route is called in situ process, but when the reinforcing phase is created or synthesized separately outside the matrix and added unto it, it is referred to as ex situ process [64]. The in situ process is usually undertaken when purity of the constituent elements is of essence. It is used when there is property specification. This method generates more homogenous dispersion of particles. Strong interfacial bonding, higher thermodynamic stability, and stronger metallurgical interaction are achieved more with an in situ process. It was observed that the Al atom segregated from Ti_2_AlN alloy during laser melting and bonded with the base metal to form Zn_7_Al [65], which was an in situ way of producing Al atoms. There was equally segregation of Ti atom from Ti_2_AlN to bond with the matrix and form Zn_7_Al-Ti_2_AlN composite, an Al- rich phase, Zn-rich phase, Ti_2_AlN, TiN, and Al_0.64_Ti_0.36_. The last two phases were aligned at the grain boundaries and enhanced its load transfer propensity [65]. This was another successful way of generating Ti atoms using the in situ process. However, the in situ process is challenged by a number of factors. Notwithstanding that the in situ process is cost effective, its commercial scalability is still poor. In addition, synthesized materials are restricted to those that are thermodynamically stable in the base material. The dynamics of nucleation and grain growth affect the particle size and configuration [66,67]. Ex situ processing, on the other hand, is more preferred to the in situ process because it is suitable for mass and bulk production, it is relatively cheaper, and properties of processed materials are dependent on the nature, grain size, and percentage quantity of the dispersed phase. However, ex situ is more prone to agglomeration of dispersed phase than in situ [68]. The agglomeration will give room to evolution of pores and weak interface bonding; even though the advantages associated with ex situ still exist. In a comparative analysis of in situ and ex situ manufacturing techniques, Kemény et al. [69] discovered that heat treatment is essential for both ex situ and in situ production of foam-filled tubes (FFTs). However, ex situ FFTs were more ductile, while the in situ FFTs had more strength because of precipitation-strengthening. The heat-treated in situ product possessed higher plateau stress and energy absorption kinetics than the ex situ product since there was tighter fitting between the foam and the tube in the in situ method; but the ex situ product had the highest compressive stress. The authors concluded that it was more advantageous to produce in situ FFTs than ex situ FFTs since the one-step manufacturing process of in situ is quicker, easier, and more cost-effective because no machining is required. However, if ex situ must be used, a further method of dispersing the reinforcement such as ball-milling should be incorporated. In addition, a heat treatment after fabrication should be conducted to enhance recrystallization which will reduce pores and refine the grains. Furthermore, development of metal matrix composites can be grouped into solid-state and liquid-state production methods. The solid-state production technique is discussed in the next subsection.

### 4.1. Solid State Production of Composites

By the application of high temperature and pressure, diffusion of atoms is achieved in solid-state consolidation of MMC or PMC. The solid-state method is usually employed for fabrication of high melting point base metals. Its advantages include reduced segregation of matrix and reinforcement, improved interfacial bonding, enhanced purity, enhanced grain refinement, reduced interfacial reaction; hence, it is used when maximum mechanical, thermal, tribological, and corrosion properties of a composite are required [70,71,72]. One of the solid-state production techniques is friction stir processing. According to Panwar et al. [73], friction stir processing (FSP) and powder metallurgy (PM) are the major processes of the solid fabrication method. FSP is used in altering the characteristics of a material via heavy localized plastic deformation. In this process, a pin is lurched into the material via the shoulder of a revolving tool adjacent to the base material. As the tool passes over the material, the revolution of the shoulder with the help of an applied load heats the material surrounding the target area and stimulates material flow which modifies the area. The microstructural evolution after FSP is a function of material flow, plastic deformation, and elevated temperature which is characterized by a central stir zone enveloped by a thermomechanically affected zone (TMAZ) and heat-affected zone (HAZ). The deformed material is conveyed from the retreating side (RS) of the tool pin to the advancing side (AS) and is forged by the tool shoulder, generating a solid state-modified material [74]. Mehdi and Mishra [75] worked on the effect of friction stir processing on microstructure and mechanical properties of tungsten inert gas (TIG) welded joint of AA6061 and AA7075 and observed that FSP enhanced the ductility of the welded joints, and that the combination of TIG and FSP gives more ductile joints than only the TIG- welded joint as a result of grain refinement accompanying FSP. The tensile strength, hardness, and percentage elongation were quite improved by the process. Babu et al. [76] researched on the effect of the tool shoulder diameter during friction stir processing of AZ31B alloy sheets of various thicknesses. It was observed that FSP was a promising technique for refinement and homogenization of grains at a chosen zone within the material. Also observed was that various defects and properties such as tensile strength of FSP is a function of the tool axial force, tool rotational speed, tool traversing speed, and tool shoulder diameter. So, when there is the need to eradicate the defects in an FSPed region, the tool shoulder diameter should be considered most. However, if the interest is more on the tensile strength and hardness, the tool traversing speed plays a major role. Defects experienced in extruded and stir-casted metal matrix composites are ameliorated with the FSP process through improvement of its microstructure when FSP is applied on such a material. However, it has been noted that some properties such as the ultimate tensile strength and yield strength are affected negatively when the feed rate is increased but hardness is independent of the feed rate [77]. 

Powder metallurgy, on the other hand, is a consolidation technique of MMC whereby powder materials are blended together with either a turbular mixer [8,78], planetary ball mill [79,80,81], vibratory ball mill [82] or any type of mixing device, followed by compressing the blend into a preferred geometry and heating in an inert environment to eliminate oxidation and contamination by surrounding air. Hence, PM entails powder-mixing, pressing to net shape, and sintering. PM is divided into two, namely, conventional and non-conventional PM techniques. Conventional PM involves cold compaction followed by sintering while non-conventional PM entails concurrent compressing and sintering [83,84]. The sintering stages comprise initial neck formation; neck growth, and densification; and the final stage characterized by pore closure and grain coarsening. Homogenous dispersion of reinforcing phases on the matrix is achieved through powder-blending. Homogeneously dispersed reinforcement is very crucial for generating good microstructure devoid of pores and enhanced mechanical properties [85]. PM has the capacity of generating accurate net shape of complicated geometry with high precision [86]. Gomez et al. [87] employed PM and hot extrusion in fabricating an Al matrix-boron carbide composite. A composite with high densification and strongly bonded matrix/reinforcement interface was generated. The effect of process parameters in Aluminium metal matrix composites consolidated via PM was studied by Vani et al. [88]. It was gathered that high sintering temperatures generated a high diffusion rate which gave rise to increased densification. In addition, appropriate selection of reinforcing phases and matrix particle sizes are essential for obtaining homogenous dispersion of the reinforcement on the matrix. Employing optimal reinforcement percentages generates a refined microstructure and finer grains. Li et al. [89] employed PM in developing titanium matrix reinforced with CNTs and graphene. It was observed that the strengthening mechanism that acted on the composite included grain refinement strengthening, carbon solid-solution strengthening, and TiC/carbon dispersion strengthening. More so, mechanical strength of the composites improved tremendously when the CNTs/Gr-dispersed phase was increased from 0.1 to 0.4 wt%. Yield strength (YS) and ultimate tensile strength (UTS) of Ti-0.4 wt% CNTs composites became improved by 40.4% and 11.4%, respectively in comparison with pure Ti. Meignanamoorthy et al. [90] disclosed that the PM technique is the easiest method for fabricating MMC with hard and soft reinforcements in comparison with other production techniques such as stir-casting, centrifugal casting, etc. Higher metallurgical bonding is recorded in the PM technique than other techniques. Stronger interfacial bonding between matrix and reinforcement is obtained with the PM method. Both mechanical and tribological properties of composites developed via PM are more improved than other techniques. Some other essential solid-state manufacturing techniques are tabulated in Table 3. 

Hot isostatic pressing (HIP) is a production method which makes use of high temperature and constant stress (isostatic) gas pressure to remove micropores and raise the density of metals, ceramics, polymers, and composite materials. Sergi et al. [101] successfully developed Ni-based MMC using hot isostatic pressing and obtained a fully densified composite and homogenous microstructure. Almotairy et al. [102] discovered that the HIP process improved the dispersion of SiC on Al matrix, and enhanced the tensile strength and microhardness of the Al-SiC composite. It was observed that the tensile stress and strain of a γ-TiAl/TiB2 composite greatly improved when it was consolidated with HIP [103]. 

**Figure 5:**
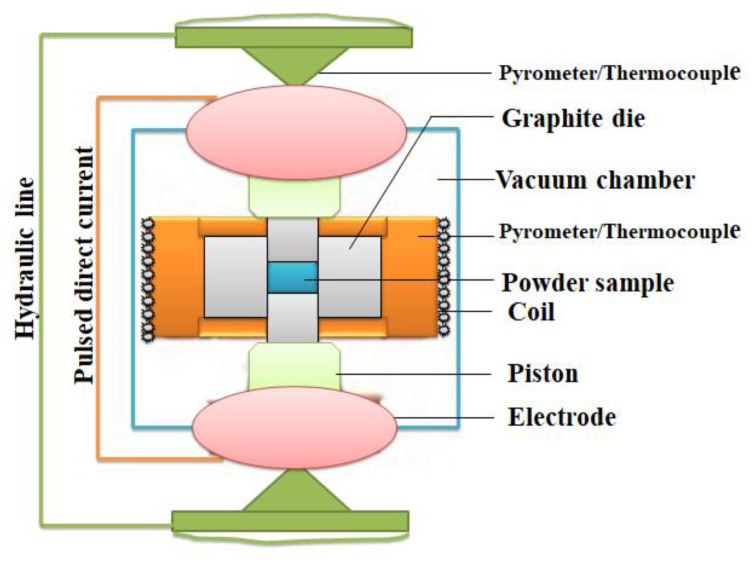
SPS Machine Adapted and Redrawn From [114].

Spark plasma sintering (SPS) has proven to be a prospective solid-state production technique for development of high-temperature, high-strength, corrosion-resistant and wear-resistant MMC and PMC because of its cost effectiveness, energy conservation, and property improvements. SPS is a classical powder metallurgy fabrication technique employed in creating very pure composites by introducing a pulsed direct current (see Figure 5) on the powder material to provoke micro- and macro-diffusion of particles to enhance densification and consolidation of powders [71,84,104,105,106]. In a comparative study of conventional sintering and SPS, Dash et al. [107] developed Cu-Al_2_O_3_ with both methods. Result showed that maximum Vickers hardness of 80 HV was obtained with conventional sintering while SPS yielded a Vickers hardness of 125 HV. In another study, SPS was compared with microwave sintering in the production of a Mg-B_4_C composite. Density, micro- hardness, and bending strength of the sample developed with SPS were higher than that produced with microwave heating. Microcracks, pores, and weak mechanical strength were more pronounced in the sample developed with microwave sintering [108]. Conventional sintering, microwave sintering, and spark plasma sintering were compared in the production of Al-TiC composite. A sample produced with SPS showed the highest density of 99%, bending strength of 291 ± 12 MPa, and hardness of 253 ± 23 HV, while conventional sintering possessed the weakest and worst properties followed by microwave sintering [109]. Gao et al. [110] observed that when the sintering temperature of the vacuum sintering technique rises to a certain temperature, the composite will possess the least porosity, best homogeneous microstructure, and highest hardness. This shows that the sintering temperature is one of the most important parameters that must be optimized if the best result is to be generated with vacuum sintering. On the other hand, microwave- sintering was reported as the better alternative to conventional sintering for large composite materials [100]. Reddy et al. [111] found out that the ductility of Al-SiC nanocomposites reduced when the volume fraction of SiC increased during the microwave-sintering fabrication technique. However, a better improvement of mechanical and thermal properties of the developed composite was recorded when it was hot-extruded after microwave-sintering. Bye and large, it can be seen that SPS is the most promising PM solid-state production technique which should be adopted in the production of TCs as attested by some other authors [112,113]. The only challenge is that this technique is yet to be developed into producing long-span products. Hence, further studies are required to equip the SPS technique with what it takes to be able to produce long-span products such as electric conductors.

### 4.2. Liquid-State Production of Composites

Liquid-state production technique of MMC or PMC generates significant characteristics of strong metallurgical bonding between the matrix and dispersed phases. In this process, the base material or matrix is usually metal with low melting temperature such as Al or polymer as it is expected to melt while the reinforcing phase is dispersed on the molten matrix. Mechanical strength/other properties of the composite are dependent on the dispersion of reinforcement as well as its nature and volume fraction. The liquid production process is more cost-effective and simpler than the solid production process besides being the process that produces complicated geometries at very fast rate; and accounts for the largest production volume on the globe [70,115,116,117]. Brittle interfacial layers and agglomeration of reinforcement on the molten matrix are some of the challenges experienced in the liquid production method [118,119]. Shirvanimoghaddam et al. [120] reported that stir-casting, squeeze-casting, and infiltration are the major liquid-state composite consolidation techniques widely adopted. Stir-casting entails melting of the matrix material, addition of dispersed phases into the molten matrix, and stirring of the mix mechanically or electromagnetically to create a vortex that pulls the dispersed phase into the molten matrix. When a homogenous mix is achieved, it is transferred into a prepared mold for solidification. It was reported that volume fraction of the dispersed phase can be as much as 40% [68,121]. An Anglo-Australian multinational company which is the world’s second-largest metals and mining corporation called Rio Tinto Incorporated uses stir-casting (Duralcan) in the development of Al380-SiC gear box components and Al359-SiC brake discs [122]. The three major classes of the liquid-state production technique are discussed below.

**Figure 6:**
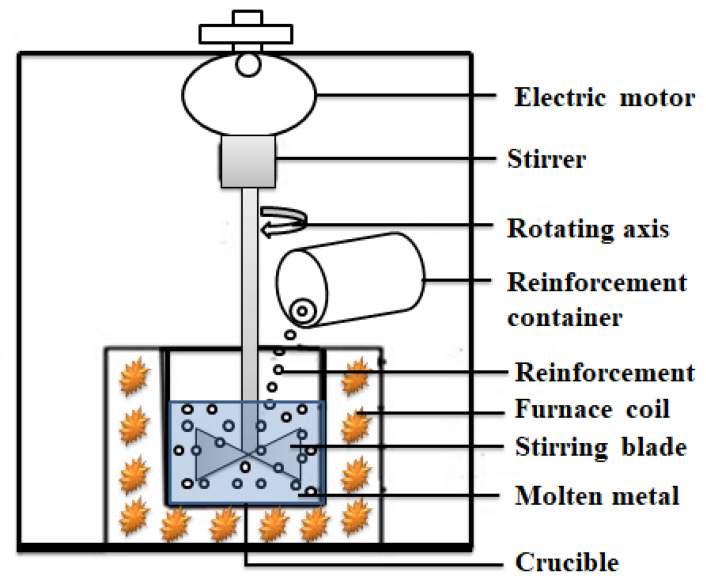
Schematic Image of Stir-Casting Process, Adapted and Redrawn From [68].

(a) Stir-casting: It is a process of producing MMC or PMC whereby the base material is heated to its molten state before the addition of the dispersed phase. The matrix and dispersed phases form the casting mixture which is stirred mechanically or electromagnetically until a uniform blend is formed, as shown in Figure 6. Then, the mixture is transferred to a mold and allowed to solidify. The merits of stir-casting are that it is very simple, flexible, and applied in mass production. However, its challenge is that a homogenous dispersion of reinforcements is difficult to achieve [123]. 

Hashim and Looney [124] stated that factors to be observed in obtaining a good product with stir-casting include: (i) Maintaining homogenous dispersion of the dispersed phase on the matrix. (ii) Ensuring full wetting out of the reinforcement by the matrix. (iii) Stirring must be uniform and slowly so as to avoid air bubbles which can generate pores. (iv) Chemical reactions of the reinforcement and matrix must be monitored and controlled to contain the evolution of unwanted phases. Bhandare and Sonawane [123] developed an Al metal matrix composite using stir-casting. The authors discovered that a uniform dispersion of reinforcement was better achieved if the stirring blade was four in number and the angle of inclination was either 45° or 60°. In addition, a higher wettability was achieved with the temperatures that converted the matrix into a semi-solid and not a complete liquid. For Al, heating to the temperature of 630 °C was recommended, so that a more homogenous dispersion of reinforcement was achieved. It was also recommended that molds should be pre-heated to reduce the porosity and improve mechanical strength. Some authors reiterated the importance of controlling the process parameters in order to obtain good stir-casted products. Such parameters as stirring temperature, stirring speed, stirring time, and preheating time must be adequately taken care of [125]. 

(b) Squeeze-casting: This is a casting method that integrates die-casting and forging. It begins with low-pressure-casting, followed by introduction of very high pressure when the cast starts cooling, to generate high-quality-casting. Squeeze-casting is a smart liquid processing method for manufacturing MMC because it generates excellent mechanical properties due to the absence of common defects such as porosity and shrinkage cavities, and the eradication of separation of the reinforcement and matrix [126,127]. This process utilizes low die-filling velocity, with smallest turbulence and high applied pressure, to create high-quality casts [128]. The two main types of squeeze-casting include direct and indirect squeeze-castings. In the direct squeeze-casting method, the pressure is applied on the whole surface of the molten composite during solidification by a punch, which generates casting with full density. However, in the indirect squeeze-casting method, the molten material is injected into the die cavity by a small-sized piston [129]. In order to obtain perfect squeeze-casted composites, homogenous dispersion of reinforcement is of utmost importance. In addition, high wettability of reinforcement by the matrix is usually achieved with the use of high pressure [130,131]. Dhanashekar and Kumar [132] suggested that the optimum pressure that should be applied in the squeeze- casting of Aluminium alloys and composites will be 100 MPa so as to obtain better microstructural refinement and improved mechanical strength. The authors equally disclosed that finer grains gave better improvement. Merits of squeeze-casting are as follows: provides wider range of shapes and components than other liquid-state methods; very minute or no machining is required in squeeze-casted products; minimal porosity; excellent surface texture; fine microstructures; enhanced strength and zero material wastes; while its challenges include: complex tooling is costly, tooling is very specific and not flexible, high precision of the process increases casting time, which increases overall cost [133]. 

(c) Infiltration: This is the process used in reducing the porosity of green sintered composites with a liquid metal or alloy that has a lower melting temperature by directing it to penetrate into the pores by means of capillary forces. It is a method in which prefabricated dispersed phases, (including particulates, fibers or ceramics) are soaked in a molten metal matrix so as to fill the pores or gaps between the reinforcement phases and the matrix. The driving force of the infiltration process may either be the capillary force of the reinforcing phase (spontaneous infiltration) or an applied pressure (vacuum, gaseous, mechanical, electromagnetic, centrifugal or ultrasonic) directed to the liquid matrix phase [134]. Pressure infiltration has advanced into commercial application such that reinforced Al-Al_2_O_3_ automobile engine components such as diesel-engine pistons, engine block cylinder liners, and crankshaft pulleys are mass-produced in Japan and Europe via pressure infiltration. Even in the United States, complexly configured Al-70vol% SiC electronic circuit substrates are being produced commercially via the pressure infiltration process; just as the tungsten-copper composite used for electrical contacts is produced with this process [135]. Proper wetting out of reinforcement is important in enhancing the quality of infiltrated composites. For aluminium matrix composites, reactive elements such as Mg, Li, Ti, Ca, and Zr are added in the matrix/reinforcement blend in order to raise the surface energy of the reinforcement, reduce the surface energy of the melt, reduce the reinforcement/molten matrix interfacial energy and so improve wetting out [136]. Another strategy for improving wetting is the use of mechanical work to force the non-wetting molten material into the preform. Even though the primary aim of applying pressure is to overcome the capillary forces, higher pressures generate additional benefits such as an increased processing rate, controlled chemical reactions, refined microstructures, and improved product quality [134]. It must be noted that applying high pressure all through, the process causes breakage or deformation of the preform. To prevent such defect, low pressure is applied when molten metal is pressed into the preform, while high pressure is applied during solidification of the composite [137,138]. 

### 4.3. Projected Materials for Production of Improved Transmission Conductor

There are two functional parts of a transmission conductor, the outer layer where electricity is conducted and the inner core that provides the framework of the conductor. Therefore, materials that are used in the outer layer must possess high electrical conductivity, low density, high corrosion resistance, high wear resistance and a low coefficient of thermal expansion (CTE). Cu and Al were the metals that possessed most of these properties mentioned above. However, Cu has high density and is not as cost-effective as Al. Therefore, Al became the mostly used material for development of the outer conducting layer of transmission conductors, though in a reinforced form because of its poor mechanical strength. Some light-weight metal matrix composites that have been researched and confirmed that they possess high mechanical, tribological, thermal, and electrical properties, and can function more creditably in the outer layer of transmission conductors include Al-Nb [7,139], Al-B_2_ [140,141], Al-CNTs [142,143,144,145], and Al-Ti alloy [146,147,148]. These composites/alloys have shown remarkable improvement in properties requisite of transmission conductors. On the other hand, the inner core requires light-weight composite materials with high creep resistance, high strength, high thermal stability, low CTE, high oxidation resistance, high wear resistance, and high corrosion resistance [5]. It is on record that conventional light-weight metal matrices such as Al, Zr, Ti have performed very well as composite matrices in wetting and compaction of various dispersed phases in MMC [149,150,151,152]. These light-weight matrices, when alloyed with carefully selected light-weight reinforcements will provide a maximum framework to transmission conductors. Reinforcements with proven mechanical, tribological, thermal, and corrosion properties which will enhance the properties of those matrices include CNTs [153,154], BN [155,156], SiC [157,158], TiC [159,160,161], B_4_C [162,163], and AlN [164,165]. Any of these materials when produced with improved techniques will perform better than the existing transmission conductor inner core in terms of mechanical strength, wear resistance, thermal expansion (sag), corrosion resistance, ampacity, and durability.

## 5. Conclusions and Recommendations

Review of papers in the open literature on the challenges bedevilling the existing transmission conductors and possible remedies to these issues has been conducted. The approach to this topic was based on accessing the factors that contribute to the poor performance of transmission conductors from both the production techniques and the materials used. So, conclusions and recommendations were based on possible remedies to these challenges.

1. Hot-rolling used in producing some Al and Cu conductors has some defects. Among the defects, scale formation is one of the most devastating defects. So, it is recommended that it can be prevented by ensuring that the scale before rolling should be thinner than the critical thickness of the work piece which is dependent on the rolling temperature. Hence, optimization of the hot-rolling temperature before conducting the actual work is highly recommended.
2. Resin additives, rpm of extruder, temperature and pressure sensors applied in extrusion technique should be optimized since the inherent defects emanate from these factors. In addition, fused filament fabrication (FFF) is a better replacement for extrusion. Furthermore, direct ink writing (DIW), which has larger scope than FFF as it can work on thermosets, thermoplastics, metals, ceramics, and cements, should be adopted too.
3. To eliminate defects in pultrusion technique, there should be uniformity and slowness of the pulling speed of the machine to avoid cracks, fibre breakages or warped products. Orifice temperature should be maintained at optimal level because it controls the structure of the pultruded product. The polymerization and rheological kinetics of the resin should be regulated by optimizing the quantity of additives and catalysts added.
4. In situ method helps improve the purity of the composite and enhance uniform dispersion of the reinforcements. However, it is not yet commercially viable. For this reason, the ex situ processing route should be adopted but dispersion should be improved via high-energy ball-milling, vacuum-sintering or SPS should be used to reduce impurities in the products, and heat treatment is encouraged to reduce pores and enhance grain refinement.
5. Solid-state fabrication technique is recommended as it enhances mechanical, thermal, tribological, and corrosion properties of composites. Meanwhile, SPS is recommended as one of the most promising solid-state methods to be adopted in developing TCs. However, it is yet to be developed for producing long-span products such as electricity wires. So, further research on SPS is recommended.
6. Liquid production process is more cost effective and simpler than solid production. It is used in producing the most complicated geometries at very fast rate. It accounts for the largest production volume on the globe. Meanwhile, stir-casting is the most versatile, simplest, and most flexible and most applied liquid production in mass production. However, its defects should be ameliorated via double step stir-casting to enhance uniform dispersion of reinforcements, coating of matrix with Cu, Mg, Ca, Li, or Ag to enhance wettability, and optimized stirring speed and temperature to avoid air bubbles and deleterious chemical reaction.
7. Projected advanced light-weight composite materials for production of the outer layer of TCs include Al-CNTs, Al-Nb, Al-Ti, and Al-B2. For the inner core, the recommended matrices included Al, Ti, and Zr; while the reinforcing phases include CNTs, BN, SiC, TiC, B4C, and AlN.
8. For selection of appropriate HTLS conductors, it is recommended that those in the tropics can adequately select ACCC since there is no or minimal ice load, while those in the temperate region should install ACCR which has more strength to withstand ice and wind loads.
9. The suggested techniques, materials, and measures if strictly followed in developing transmission conductors would promote a sustainable steady supply of electricity in the grids, which will fast-track growth and development in the third world countries and sub-Saharan Africa.

## Figures and Tables

**Table 1:** HTLS Conductors and their Properties.

No.	HTLS	Outer Layer	Core	Strengths	Deficiencies	Refs.
1	ACSS	Annealed 1350-O Al	Steel	High strength, high operating temperature (250 °C)	High corrosion rate, high density, high CTE	[5,20]
2	G-TACSR	Trapezoid Al wire	Steel	High strength, high operating temperature (210 °C)	High corrosion rate, high density, high CTE	[21]
3	ACCR	Al-Zr wire	Al_2_O_3_ fibre	High strength, high operating temperature (210 °C), low corrosion rate, lower CTE than steel-based	Relatively high CTE	[22,23,24]
4	ACCC	Annealed 1350-0 Al	C-epoxy	Very low CTE, low density, average temperature (130 °C)	Low strength	[6,14,25,26]
5	Z-TACIR	Annealed Al	Invar (64 steel, 34 Ni)	High operating temperature, high corrosion resistance	Strength lower than steel, high density	[14,20]

Key: ACSS—aluminium conductor steel supported, ACCR—aluminium conductor composite reinforced, ACCC—aluminium conductor composite core, G-TACSR—Gap-Type aluminium conductor steel reinforced, Z-TACIR—Zirconium-type aluminium conductor invar reinforced, CTE—coefficient of thermal expansion.

**Table 2:** Defects Common with Extrusion Technique [18].

No.	Extrusion Defects	Manifestation on Product	Remedies/Prevention
1	Inappropriate installation	Marks on the product	Adjust the die setting; check for alignment
2	Inappropriate operation	Irregular wall thickness	Use digital pressure/temperature sensors
3	Resin faults	Indentations on the products	Precision in resin addition
4	Inappropriate addition of materials	Formation of bubbles	Precision in calculation/addition of materials
5	Surging	Irregular thickness of products	Running the extruder gentler or quicker by 10%
6	Inadequate mixing	Clogging in the products	Raise the mixing speed back pressure
7	Melt fracture	Rough surface	Apply correct additives; keep to the rpm of extruder
8	Overheating	Irregular cooling causing warping	Quench the barrel heat except in the rear side; cool the barrel if required
9	Moisture release	Pits, long bubbles and dotted lines	Material must be pre-dried; use vent in the extruder
10	Trapped air	Dotted lines, pits and bubbles	Shun extruder over speed

**Table 3:** Further Solid-State Production Technique.

S/N	Production Method	Merits	Demerits	Refs.
1	Spark plasma sintering	(i) Low energy consumption (ii) Refined microstructure (iii) Short sintering time (iv) Low grain growth (v) Improved properties (vi) Purified products	(i) Only simple shapes are produced (ii) Pulsed DC generator is expensive (iii) High cost of equipment	[84,91,92,93]
2	Hot isostatic pressing	(i) Removes porosity (ii) Heating and pressing at a single step (iii) Diffusion bonding (iv) Fine grains	(i) Expensive tooling (ii) Lower yield strength (iii) Longer processing time	[94,95,96]
3	Cold isostatic pressing	(i) Cheap tooling (ii) Uniform density (iii) High green strength (iv) Hard to press materials	(i) Dimensional inconsistency (ii) Additional machining is costly (iii) Longer processing time	[94,96]
4	Diffusion bonding	(i) Similar and dissimilar materials can be joined (ii) Joint formed is pure, clean and devoid of pores (iii) Similar physical and mechanical properties of base materials and produced composite (iv) Reduced plastic deformation	(i) Time consuming (ii) Not suitable for mass production (iii) Set up cost is high	[97,98]
5	Vacuum sintering	(i) Highly controllable (ii) Large scale production (iii) Fewer defects (iv) Pure products	(i) High cost of tooling (ii) High cost of raw materials.	[99]
6	Microwave sintering	(i) Low energy consumption (ii) high heating rate (iii) Short sintering time (iv) High densification	(i) High initial cost (ii) Large quantity of materials are required	[100]

## Data Availability

Not applicable.
